# Increased Muscle Stress-Sensitivity Induced by Selenoprotein N Inactivation in Mouse: A Mammalian Model for *SEPN1*-Related Myopathy

**DOI:** 10.1371/journal.pone.0023094

**Published:** 2011-08-08

**Authors:** Mathieu Rederstorff, Perrine Castets, Sandrine Arbogast, Jeanne Lainé, Stéphane Vassilopoulos, Maud Beuvin, Odile Dubourg, Alban Vignaud, Arnaud Ferry, Alain Krol, Valérie Allamand, Pascale Guicheney, Ana Ferreiro, Alain Lescure

**Affiliations:** 1 Architecture et Réactivité de l'ARN, Université de Strasbourg, CNRS, IBMC, Strasbourg, France; 2 UPMC Univ Paris 06, IFR14, Paris, France; 3 CNRS, UMR7215, Paris, France; 4 Inserm, U974, Paris, France; 5 Institut de Myologie, Paris, France; 6 Inserm, UMR U787, Paris, France; 7 UPMC Univ Paris 06, site Pitié-Salpêtrière, Département de Physiologie, Paris, France; 8 Université Paris Descartes, Paris, France; 9 Inserm, U956, Paris, France; 10 AP-HP, Groupe Hospitalier Pitié-Salpêtrière, Consultation des Maladies Neuromusculaires, Paris, France; Johns Hopkins University School of Medicine, United States of America

## Abstract

Selenium is an essential trace element and selenoprotein N (SelN) was the first selenium-containing protein shown to be directly involved in human inherited diseases. Mutations in the *SEPN1* gene, encoding SelN, cause a group of muscular disorders characterized by predominant affection of axial muscles. SelN has been shown to participate in calcium and redox homeostasis, but its pathophysiological role in skeletal muscle remains largely unknown. To address SelN function *in vivo*, we generated a *Sepn1*-null mouse model by gene targeting. The *Sepn1^−/−^* mice had normal growth and lifespan, and were macroscopically indistinguishable from wild-type littermates. Only minor defects were observed in muscle morphology and contractile properties in SelN-deficient mice in basal conditions. However, when subjected to challenging physical exercise and stress conditions (forced swimming test), *Sepn1^−/−^* mice developed an obvious phenotype, characterized by limited motility and body rigidity during the swimming session, as well as a progressive curvature of the spine and predominant alteration of paravertebral muscles. This induced phenotype recapitulates the distribution of muscle involvement in patients with *SEPN1*-Related Myopathy, hence positioning this new animal model as a valuable tool to dissect the role of SelN in muscle function and to characterize the pathophysiological process.

## Introduction

Selenium is of fundamental importance to human and animal health. Dietary restriction of this trace element pointed to its implication in cancer prevention, viral infections protection, fertility and aging [1], [2]. The major biological form of selenium is selenocysteine, the 21^st^ amino acid, which is co-translationally incorporated into a specific class of proteins (selenoproteins), through a dedicated machinery [3]. The presence of this particular amino acid in seleno-enzyme active sites confers an increased catalytic reactivity due to the particular redox and nucleophilic properties of the selenol group [4], [5]. In addition to the above-mentioned dysfunctions, it was shown that selenium deficiency, sometimes associated with simultaneous lack of vitamin E, causes different muscular pathologies (reviewed in[6], [7]). Since 2001, mutations in the human *SEPN1* gene encoding selenoprotein N (SelN) have been associated with four early-onset, autosomal recessive neuromuscular disorders, now recognised to represent a unique disease termed *SEPN1*-Related Myopathy (*SEPN1*-RM) [8]–[11]; reviewed in [12]. This muscular disease is clinically homogeneous, characterized by generalized muscle atrophy and predominant weakness of neck and trunk muscles from infancy, leading to severe scoliosis, spinal rigidity and life-threatening respiratory insufficiency in childhood or adolescence, but contrasting with relatively unaffected limb strength and ambulation. On the other hand, the histopathological presentation is heterogeneous with most *SEPN1*-RM muscle biopsies showing predominance and/or hypotrophy of type I fibers, as well as small focal areas of mitochondria depletion and sarcomere disorganization (short core lesions or “minicores”) within muscle fibers; protein aggregates have been observed in some cases but are less frequent, and necrosis/regeneration is mild or absent [12]. Many *SEPN1* mutations have been described so far, but no direct phenotype-genotype correlation could be established [7], [13]–[15]. This observation, together with the large morphological spectrum of *SEPN1*-RM, suggests a complex underlying pathophysiological mechanism.

Cellular and biochemical studies established that SelN is a trans-membrane glycoprotein of 65-kDa located in the endoplasmic reticulum. Examination of the amino acid sequence identified a putative calcium binding EF-hand motif next to a membrane-addressing signal peptide at the N-terminus, but no function could be proposed based on sequence comparison or homology search [16]. Recent experiments revealed physical and functional interaction between SelN and the ryanodine receptor (RyR), a calcium release channel of the sarcoplasmic reticulum [17]. In zebrafish embryos or patient muscles depleted in SelN, RyR displayed reduced activity and abnormal sensitivity to redox conditions [17]. Therefore, it was proposed that SelN regulates calcium homeostasis by modulating RyR redox status. Moreover, recent *in vitro* studies demonstrated that SelN plays a role in cell defense against oxidative stress; absence of SelN in human cells resulted in a significant increase in basal intracellular oxidant activity and protein oxidation, as well as an increased susceptibility to exogenous oxidative stress [18]. The conclusion of these experiments positions SelN as a key regulator of cell stress, redox signaling and calcium homeostasis pathways (reviewed in [12]).

Although *Sepn1* mRNA and SelN protein were detected in almost all human and mouse tissues [19], [20], its deficiency is associated with a muscle-specific dysfunction in humans. Interestingly, SelN expression was higher in embryonic tissues compared to adult. Furthermore, *in vitro,* its expression decreased during myoblast differentiation [16]. In zebrafish and mouse embryos, early embryonic expression of *Sepn1* was reported to be mostly abundant in somites, the precursors of muscle structures, diminishing throughout muscle differentiation [20], [21]. This expression pattern is in favor of a role for SelN in muscle development. Consistently, morpholino-mediated inhibition of the *sepn1* gene expression in zebrafish caused marked developmental defects in somite organization, establishment of slow fibers and muscle architecture, impairing embryo motility [17], [22].

To determine the role of SelN *in vivo* and to analyze the implications of its deficiency, we developed a *Sepn1* knock-out mouse model that constitutes the first mammalian model for *SEPN1*-RM. We recently showed that SelN deficiency does not alter somitogenesis or the expression of myogenic factors in this model. This result contrasted with those obtained in zebrafish, suggesting that SelN is dispensable for normal embryogenesis in mouse [20]. Here, we demonstrate that SelN-deficient adult mice do not spontaneously develop muscle disorders and are indistinguishable from wild-type, although subtle muscle alterations could be detected. Hence, effect of *SEPN1* mutation is markedly different in mouse and human, limiting the use of these *Sepn1^−/−^* mice as a clinical model for *SEPN1*-RM. Importantly, mutant mice display increased susceptibility to swimming-induced exercise and stress; under these conditions, *Sepn1* mutant mice developed muscle atrophy, predominantly affecting trunk muscles and leading to severe kyphosis. These features are reminiscent of the axial weakness distribution and scoliosis characteristic of *SEPN1*-RM patients. Therefore, we propose that this model constitutes a useful tool to clarify the role(s) of SelN, to better understand the pathophysiological mechanisms underlying *SEPN1*-RM and the influence of environmental factors in the severity and progression of these muscular disorders.

## Results

### Excision of *Sepn1* exon 3 disables SelN expression, but leads to no obvious macroscopic phenotype in the *Sepn1*-null mice

**Figure 1:**
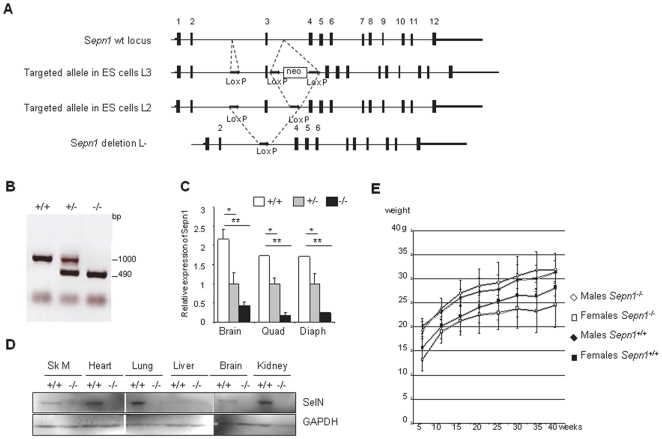
Generation of the *Sepn1* knock-out mouse model. **A**) Strategy for the selective excision of *Sepn1* exon 3. In the targeting fragment, LoxP sites were incorporated on each side of exon 3 together with a floxed neomycin (neo) cassette (L3). This fragment was electroporated into ES cells and neo-resistant clones that featured homologous recombination were selected. *In vivo* or *ex vivo* Cre-induced excision of floxed regions leading to neo excision (L2) or complete excision (L-) are represented. **B**) Mice genotyping. PCR were performed on genomic DNA extracted from *Sepn1^+/+^, Sepn1^+/−^* and *Sepn1^−/−^* mice. Exon 3 excision leads to a 500 bp loss in the PCR product size. The bands at 1000 and 490 bp represent the wild-type and the exon 3-deleted alleles, respectively. **C**) Expression of *Sepn1* transcript in *Sepn1^+/+^, Sepn1^+/−^* and *Sepn1^−/−^* tissues. Brain, quadriceps (quad) and diaphragm (diaph) were tested. Quantification was performed by qRT-PCR and normalized to the *18S* gene expression. n = 3, *, p<0.05; **, p<0.01. **D**) Selenoprotein N (SelN) expression. Western blot analysis was performed using total protein extracts from several tissues (Sk M, skeletal muscle) of *Sepn1^−/−^* and *Sepn1^+/+^* mice. GAPDH was used as a loading control. **E**) Body weight analysis. Body weight evolution of wild-type or mutant, male or female mice, from 5 to 40 weeks of age was monitored. Similar growth curves were observed for mutant and control mice of both genders.

A 7 kb genomic fragment, spanning exons 1 to 5 of the murine *Sepn1* gene, was obtained by screening a mouse 129 Sv Pas genomic library. Heterozygous (L_3_/+) and (L_2_/+) *Sepn1*-floxed 129 Sv ES cell lines were generated using a gene-targeting vector in which *loxP* sites were inserted to flank the murine exon 3 (Figure 1A). The obtained ES cells were injected into C57BL/6J blastocysts. Splicing of the modified *Sepn1* mRNA after Cre-mediated excision resulted in a frameshift downstream of exon 2 and introduced several stop codons within exon 6. *Sepn1* knock-out animals were obtained by breeding L_3_/L_3_ floxed mice with mice expressing Cre ubiquitously under the control of the CMV minimal promoter, leading to *Sepn1* gene disruption in the germinal lineage and transmission of the mutant allele to offspring. The resulting progenies were genotyped by PCR using primers that specifically targeted the deleted exon 3 (Figure 1B).

*Sepn1* transcript expression in *Sepn1^+/+^, Sepn1^+/−^* and *Sepn1^−/−^* littermate mice was quantified by qRT-PCR in several isolated tissues. We observed an almost complete disappearance of the mRNA in homozygous tissues, while its expression was reduced by two-fold in heterozygous, compared to wild-types (Figure 1C and data not shown). These results showed that removal of the third exon led to the destabilization and degradation of the *Sepn1* transcript, most likely due to the occurrence of premature stop codons. Accordingly, SelN expression was undetectable in *Sepn1^−/−^* mice by Western blot using protein extracts from different tissues, while it was clearly observed in all wild-type tissues, although at different levels (Figure 1D). These results verify that, as expected, excision of exon 3 constitutively disabled SelN synthesis in all tissues.

Out of 123 pups born from heterozygous *Sepn1^+/−^* intercrosses, 32 were *Sepn1^+/+^* (26%), 58 *Sepn1^+/−^* (47.2%) and 33 S*epn1^−/−^* (26.8%). These results are consistent with Mendelian ratios suggesting that SelN is dispensable for normal embryonic development in mice. This observation is also in agreement with our previous results demonstrating the lack of embryonic developmental phenotype [20]. Moreover, *Sepn1^−/−^* mice were healthy, fertile, and appeared indistinguishable from wild-type and heterozygous littermates from birth to adult stages. No significant difference in growth rate was observed between the two groups (Figure 1E). *Sepn1^−/−^* and control mice had a similar life expectancy, *Sepn1^−/−^* individuals reaching adulthood without developing any specific phenotype. Macroscopic and histological examination of several non-muscle organs (see Material & Methods) revealed no obvious anomalies in the *Sepn1*-null mice (data not shown).

SelN-deficient mice were ambulant and displayed no reduction of spontaneous activity. Functional and behavioral tests showed no significant differences compared to wild-types: *Sepn1^−/−^* mice exhibited no perturbations in their running performance, even after repeated treadmill training (see Data S1), and adapted to the rotarod test as efficiently as controls. These results demonstrate that SelN is dispensable for post-natal development, growth and normal activity in this model under standard breeding conditions. Similar results were obtained with the muscle-specific knockout mice obtained by crossing *Sepn1* L_2_/L_2_ with Actin-Cre mice (data not shown). Taking into account the conservative phenotype, we next focused our attention on the ubiquitous knock-out strain.

### Muscle structure and function show only subtle changes in *Sepn1* knock-out mice under basal conditions

A comprehensive analysis of *Sepn1^−/−^* muscle tissue was performed. Relative to body weight, individual skeletal muscle masses from 3 and 10 month-old mutant mice were not statistically different from control, except for the tibialis anterior (TA) muscle which was significantly reduced at both ages (wild-type: 0.164+/−0.002%, *Sepn1^−/−^*: 0.151+/−0.003% at 3 months, p = 0.0028; wild-type: 0.177+/−0.003%, *Sepn1^−/−^*: 0.147+/−0.005% at 10 months, p = 0.015). Maximal force production was measured *in situ* in TA muscles from 3 month-old mice and revealed a 15% reduction in the absolute maximal force (P_0_) in *Sepn1^−/−^* females compared to controls, while the specific maximal force (sP_0_) was unchanged (Table 1). These results indicate that hindlimb muscle contractility was not markedly affected in the absence of SelN.

**Table 1:** Contractile properties of tibialis anterior (TA) and diaphragm muscles, in basal conditions and after FST.

	*Males*		*Females*	
	*Sepn1^+/+^*	*Sepn1^−/−^*	*Sepn1^+/+^*	*Sepn1^−/−^*
TA				
Mass (mg)	57.4±1.8	53.8±2.5	38.5±1.2	37.8±1.3
P_0_ (mN)	1132.8±79.4	1227.7±94.8	867.5±33.6	741.5±30.7^a^
sP_0_ (N/g)	19.7±0.8	22.9±0.9	22.6±0.9	19.8±1.3
TA – FST				
Mass (mg)	55.4±3.3	43.8±1.9^b^	39.6±1.3	36.7±1.6
P_0_ (mN)	1167.5±74.4	957.2±47.5^a^	801.4±38.5	703.6±62.3
sP_0_ (N/g)	21.1±0.6	21.8±0.7	20.4±1.0	19.1±1.3
F30% (s)	32.5±1.8	33.9±1.9	29.8±5.0	29.1±3.7
Diaphragm – FST				
sP_0_ (N/mm^2^)	168.7±13.5	135.1±10.1		
F30% (s)	38.1±1.3	42.5±4.5		

P0, absolute maximal force; sP0, specific maximal force; F30%. time for initial maximal force to fall by 30%. Statistical comparison was performed using a Student t test. Group in basal conditions: n>6 for females. n>3 for males. Group with FST: n>7 for TA females. n>9 for TA males. n>5 for diaphragm. Statistical analysis:

a P<0.05.

b P<0.01 compared to wild-type.

**Figure 2:**
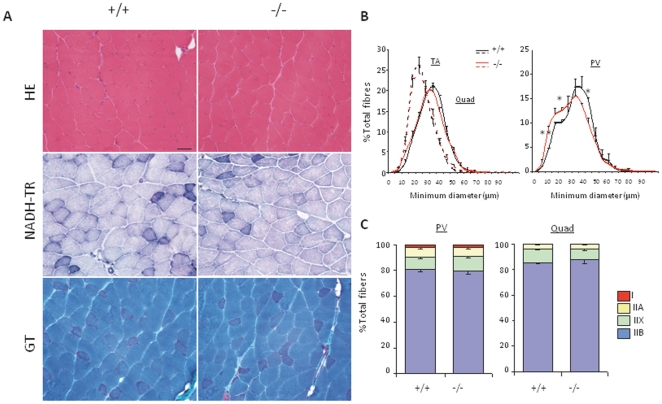
Histomorphology of *Sepn1^−/−^* adult muscles. **A**) Representative image of quadriceps cryosections, stained with HE, NADH-TR and GT, from 10 month-old mutant and control mice. Mutant muscle displayed no obvious histopathological abnormalities. Scale bar, 50 µm. **B**) Fiber size distribution (% of total number of fibers) according to their minimum diameter (µm) for TA and quadriceps muscles (left panel) and paravertebral (PV) muscles (right panel) from 10 month-old mutant and wild-type mice. A switch toward smaller fibers was observed in *Sepn1^−/−^* PV muscles compared to wild-types. n = 3, * p<0.05; ** p<0.001. **C**) Fiber type distribution (% of total number of fibers) based on type I and II MHC immunostaining of paravertebral and quadriceps muscles, from 10 month-old mice. No significant difference was found between mutant and control mice. n = 3. Data are means±SEM.

Standard histological analyses were performed on hindlimb and paravertebral muscles, as well as diaphragm, from 1, 6 and 9–12 month-old mice. At all ages studied, haematoxylin and eosin (HE) staining revealed normal muscle organization, with no sign of necrosis/regeneration, inflammation or endomysial fibrosis in any of the mutant muscles (Figure 2A and data not shown). The percentage of central nuclei was not increased in mutant mice at any of the ages studied, compared to wild-types. Moreover, reduced nicotinamide adenine dinucleotide-tetrazolium reductase (NADH-TR), succinate dehydrogenase (SDH) and cytochrome c oxidase (COX) staining showed an identical distribution of sarcoplasmic reticulum (SR) and mitochondria in wild-type and *Sepn1^−/−^* muscles (Figure 2A). In *Sepn1^−/−^* males, modified gomori trichrome (GT) staining detected focal aggregates in less than 1% of the fibers in TA and quadriceps, which were similarly observed in wild-type littermates. Periodic acid Shiff (PAS) and oil Red O (ORO) colorations revealed no change in the glycogen and lipid content of mutant muscles compared to wild-types (data not shown).

Based on immunostaining of the different myosin heavy chain (MHC) isoforms, we also demonstrated that embryonic MHC positive fibers were detected only rarely in *Sepn1*-null adult muscles, consistent with the absence of necrosis/regeneration events (data not shown). Measurement of fiber minimum diameter, based on laminin immunostaining, at 10 months of age in TA and quadriceps muscles showed similar fiber size distribution between mutant and control mice of the two groups (Figure 2B). In contrast, a slight shift toward smaller fibers was observed in the paravertebral muscles (Figure 2B). Fibers of types I, IIA, IIX and IIB in hindlimb and back muscles were in equal proportions between mutant and control mice (Figure 2C).

**Figure 3:**
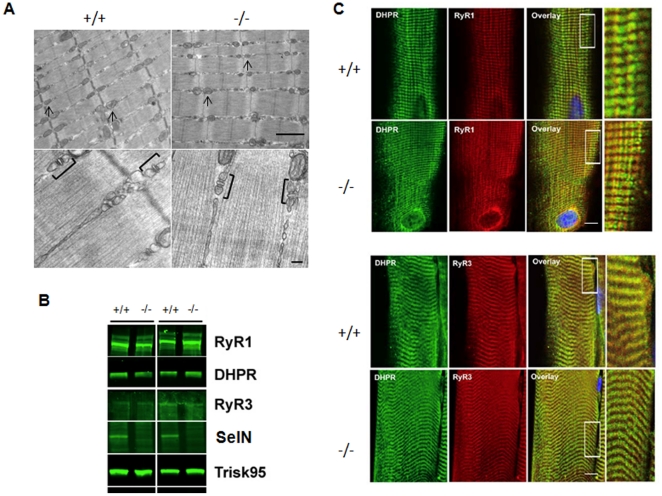
Ultrastructural organization and triadic junctions in *Sepn1^−/−^* adult muscles. **A**) Electron microscopy image of gastrocnemius muscles from 6 month-old wild-type and mutant mice. No defect in sarcomere organization, mitochondria morphology (arrows) and triads structure (brackets) were observed in mutant (right panel), compared to wild-types (left panel). Scale bars, 1 µm (top) and 100 nm (bottom). **B**) Immunostaining for DHPR (green) and RyR1 or RyR3 (red) on longitudinal sections of quadriceps from 2 month-old mice, revealing normal localization of both receptors in mutant mice compared to controls. Nuclei are revealed by DAPI staining. Boxed regions in the merged images are magnified as inserts (right) showing intracellular co-localization (yellow). Scale bar is 10 µm. **C**) Skeletal muscle microsomes extracts from *Sepn1* ^+/+^ or *Sepn1* ^−/−^ littermates were immunoblotted for Ryanodine Receptor type 1 (RyR1), Ryanodine Receptor type 3 (RyR3), Dihydropyridine Receptor (DHPR), Selenoprotein N (SelN) and triadin isoform Trisk 95 (Trisk 95).

To proceed further, electron microscopy (EM) analyses were performed on quadriceps and gastrocnemius muscles from 1 and 6 month-old mice. At both ages, EM studies disclosed a global normal ultrastructure in terms of sarcomere organization, mitochondria distribution or morphology, nuclei appearance and myotendinous junction structure (Figure 3A). No core lesion was observed. Moreover, the SR and T-tubules, including triads, appeared correctly arranged in mutant muscles (Figure 3A). Because of the proposed interaction between SelN and the ryanodine receptor RyR [17], expression levels and localization of these channels were investigated in the *Sepn1^−/−^* muscles. Western blot analysis showed that expression levels of RyR1 and RyR3, as well as other components of the triadic junction, the dihydropyridine receptor (DHPR) and the triadin isoform Trisk95, were not modified in the SelN-deficient muscles (Figure 3B). Immunostaining for RyR1, RyR3 and DHPR revealed normal intracellular distribution and colocalization at the triad sites (Figure 3C).

Taken together, these data revealed that SelN-deficient mouse muscles displayed only subtle abnormalities (TA atrophy and mild decrease in the absolute maximal tetanic force, reduced fiber size diameter in paravertebral muscles) under normal housing conditions.

### Expression levels of other selenoproteins are not modified in *Sepn1^−/−^* mouse

In light of this lack of obvious phenotype in SelN-deficient mice, the expression of several other selenoproteins was quantified in isolated tissues (brain, diaphragm and quadriceps) from mutant mice and compared to wild-types. We chose to quantify their expression levels at late embryonic stages because of the high expression of SelN at this stage. No significant difference was detected in the transcript expression levels of these proteins, except for selenoprotein R (SelR) which appeared increased in *Sepn1^−/−^* quadriceps, although to a limited extent, 1.5 fold (Figure S1). These results suggest that no functional compensation between selenoproteins occurred.

### Stress conditions trigger a pathological phenotype in *Sepn1* knock-out mice

**Figure 4:**
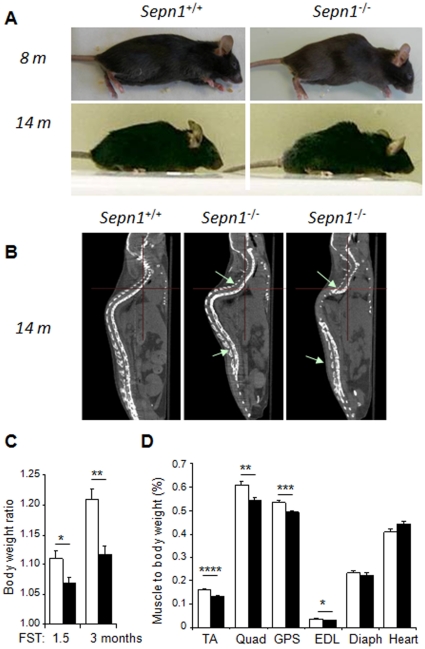
Global phenotype of *Sepn1^−/−^* mice submitted to repeated forced swimming tests (FST). **A**) Global appearance of 8 and 14 month-old wild-type and mutant mice, after 3 months of FST. A severe kyphosis was observed in both young and older mutant mice, while no sign of kyphosis was present in wild-type animals. **B**) Sagittal tomography of wild-type and mutant mice placed in ventral position, indicating a marked axial distortion of the spine in *Sepn1* ^−/−^ animals (arrows). **C**) Gain of body mass for wild-type and mutant mice, after 1.5 and 3 months of FST. The gain was significantly lower in mutant mice, compared to wild-types, at both stages. n = 20 *Sepn1* ^+/+^ *vs.* 22 *Sepn1* ^−/−^ for 1.5 months, n = 14 *Sepn1* ^+/+^ *vs*. 22 *Sepn1* ^−/−^ for 3 months. * p<0.01, ** p<0.0005. **D**) Muscle masses normalized to body weight (%) in wild-type and mutant mice after 3 months of FST. Atrophy was observed for TA, quadriceps (Quad), gastrocnemius/plantaris/soleus muscles group (GPS) and extensor digitorum longus (EDL) muscles from mutant mice, compared to wild-types. Diaphragm (Diaph) and heart masses were not affected. n = 11 *Sepn1* ^+/+^ *vs*. 16 *Sepn1* ^−/−^ (except for diaphragm: n = 8 *Sepn1* ^+/+^ *vs*. 10 *Sepn1* ^−/−^), * p<0.05, ** p<0.001, *** p<0.0005, **** p<0.0001. Data are means±SEM.

Mice were submitted to repeated forced swimming tests (FST), which, in addition to the increased physical activity, simultaneously provides a global stress context resulting in a combination of environmental, respiration and temperature stresses. Two groups of mice, aged 4–5 and 12 months, were subjected to up to 15 minutes of active swimming, every other day for three months. During the training period, *Sepn1^−/−^* mice adapted to the test similarly to wild-types. However, after four to six weeks, most *Sepn1^−/−^* mice developed, during and following the swimming exercise, an obvious phenotype characterized by a progressive rigidity of the trunk and limbs and a drastic reduction in motility (see Movie S1). There was some degree of variability in the severity and time of appearance of the phenotype among different knock-out animals. In the final stages, body rigidity persisted for several minutes after the swimming period, after which the mice recovered their normal mobility. In addition, mutant mice developed a severe kyphosis that worsened with repeated FST and was more marked in older mice; this kyphosis was initially transient and evolved progressively to an abnormal, permanent, “crouched” attitude (Figure 4A). Tomographic investigation showed a marked curvature of the spine in its central part (Figure 4B). The kyphotic angles (Th7-L4) were measured in older mice using the Cobb method [23]. The thoracic angle was around 76 degrees for control mice, increasing to 100–110 degrees in *Sepn1^−/−^* mice. These results show that SelN-deficient mice display an enhanced susceptibility to physical exercise when performed under stress conditions, such as FST.

Following 1.5 months of FST, mutant mice from the younger group exhibited a reduced gain in body weight compared to wild-types (Figure 4C). Furthermore, masses of TA, quadriceps, gastrocnemius and extensor digitorum longus (EDL) muscles, normalized to the body weight, were significantly reduced in *Sepn1^−/−^* mice, compared to controls (Figure 4D). No difference was detected for diaphragm and heart. In older mice, paravertebral muscles appeared macroscopically atrophic, while hindlimb muscles were relatively preserved, and tomography imaging confirmed an atrophic aspect of the trunk (Figure S2). *In situ* measurement of the absolute maximal tetanic forces revealed a significant 19% reduction of TA muscles from mutant males, whereas it was decreased to a lesser extent in females from the younger group. In both genders, the specific maximal force of TA and diaphragm was preserved, indicating that contractility of the fibers was unaltered (Table 1). TA and diaphragm response to fatigue, represented by the F30%, was not affected in mutant mice submitted to FST (Table 1).

**Figure 5:**
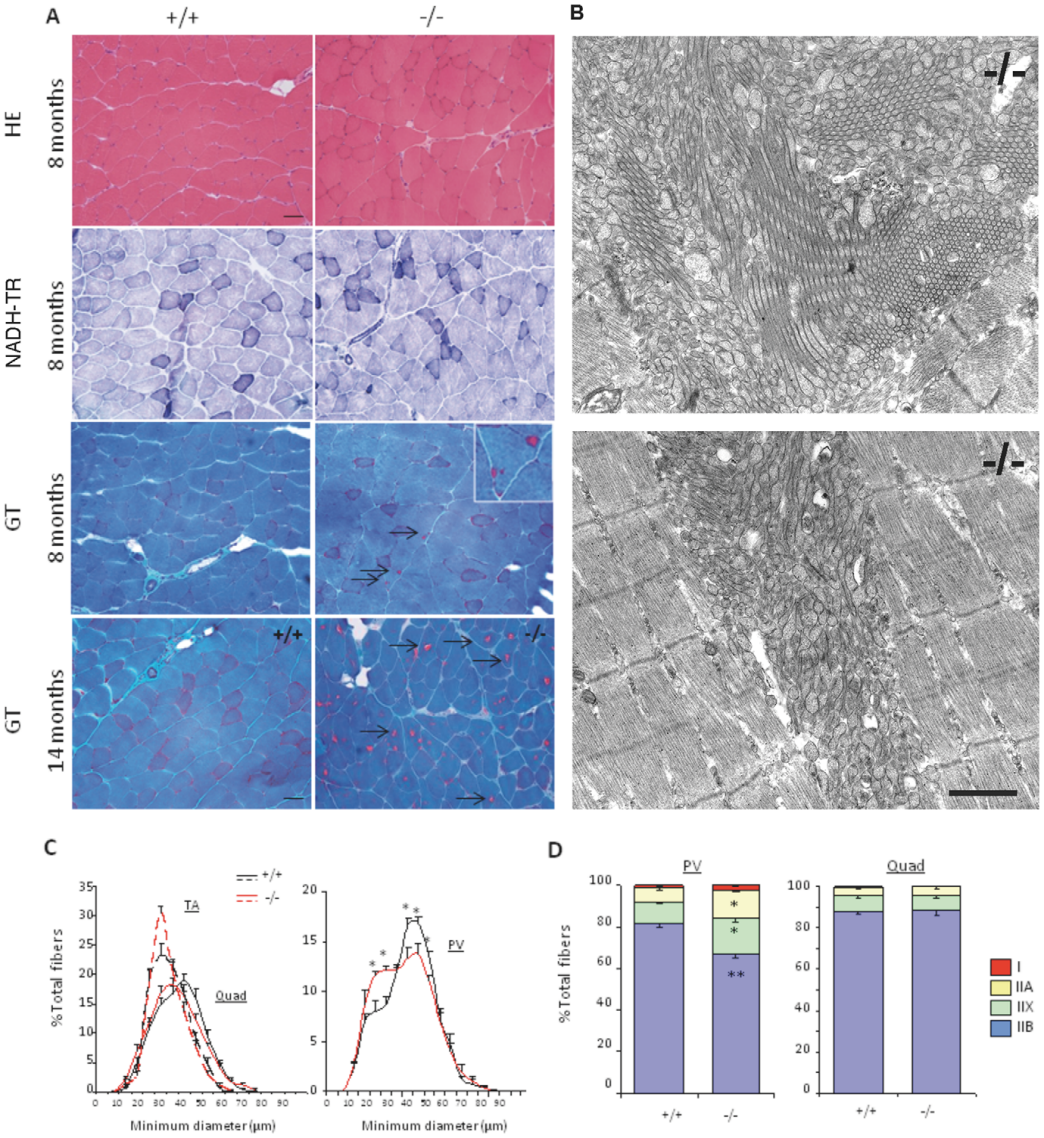
Histomorphology of *Sepn1^−/−^* muscles after 3 months of FST. **A**) Representative images of quadriceps sections, stained with HE, NADH-TR and GT, from 8 and 14 month-old mice submitted to 3 months of FST, as indicated. Cytoplasmic inclusions, stained in red by GT (arrows), were detected in several muscle fibers from young mice, and were much more abundant in old mutant mice. Aggregates were only occasionally observed in *Sepn1* ^+/+^ mice. Scale bar, 50 µm. **B**) Representative images of tubular aggregates observed in mutant quadriceps (left panel) and gastrocnemius (right panel) from old mutant mice, after FST, by electron microscopy. Accumulation of sarcoplasmic reticulum membranes was observed in the majority of mutant fibers, leading to large aggregates inclusions in the cytoplasm. Scale bar, 1 µm. **C**) Fiber size distribution, according to their minimum diameter (µm), for TA and quadriceps muscles (right panel) and paravertebral muscles (left panel) from 8 month-old mice, at the end of the test. Selective specific switch towards smaller fibers was observed in mutant paravertebral muscles, while no major alteration was detected for other muscles. n = 4 *Sepn1* ^+/+^ *vs.* 6 *Sepn1* ^−/−^, * p<0.05. **D**) Fiber type repartition (% of total number of fibers) based on type I and II MHC immunostaining for paravertebral and quadriceps muscles, from 8 month-old mice, after FST. Significant switch from type IIB to types IIX and IIA was only observed for paravertebral muscles in mutant mice, compared to wild-types. n = 4 *Sepn1* ^+/+^ *vs*. 6 *Sepn1* ^−/−^, * p<0.005, ** p<0.001. Data are means±SEM.

Histological analyses after FST revealed absence of necrosis, regeneration, endomysial fibrosis or cores in both mutant and wild-type muscles (Figure 5A); the distribution of oxidative activity was comparable in both groups and identical to basal conditions. Only in one 12 month-old *Sepn1^−/−^* animal, the most affected one, an increased number of centrally located nuclei (25% of fibers) was observed in paravertebral muscles, which were atrophic. In the younger group (7–8 month-old at the end of the test), few large tubular aggregates were detected by modified Gomori Trichrome (GT) coloration in quadriceps and back muscles in mutant mice, while only rare ones could be observed in wild-types (Figure 5A). These tubular aggregates were much more abundant in older mice and present in both female and male *Sepn1^−/−^* mice. Histochemical and EM analyses revealed their presence in a majority of fibers from mutant quadriceps, gastrocnemius, TA and, to a lesser extent, soleus and paravertebral muscles (Figure 5A and 5B). Immunohistochemical studies showed accumulation of triadic junction proteins including RyR1, RyR3, triadin and DHPR in these aggregates, suggesting a SR origin (Figure S3). Determination of the fiber size distribution demonstrated that the fiber hypotrophy observed in mutant back muscles (Figure 2B) worsened as a consequence of FST (Figure 5C). Immunostaining against different MHC revealed an important switch in the fiber type distribution in *Sepn1^−/−^* paravertebral muscles, from type IIB to types IIX and IIA, compared to wild-types (Figure 5D). In the mutant hindlimb muscles, only a limited shift toward smaller fibers and no alteration in fiber types were observed (Figure 5C and 5D).

Taken together, these results demonstrated that *Sepn1^−/−^* mice are indeed affected by swimming-induced exercise and stress, leading to kyphosis and predominant alteration of back muscles. In addition, we observed a switch toward slower/smaller fibers and the accumulation of tubular aggregates.

## Discussion

*SEPN1*-Related Myopathy describes a group of muscle diseases, clinically homogeneous although with variable severity, all caused by mutations in the gene encoding selenoprotein N (SelN). The main characteristic features are prevalent atrophy and weakness of trunk muscles, which are present from infancy but lead to complications such as scoliosis and respiratory failure later in life. To date, no curative treatment is available, and the pathophysiological mechanisms remain largely unknown. In this context, development of animal models reproducing the biochemical defect of the human condition is an important step. In this report, we describe for the first time a mammalian model for SelN deficiency, obtained by inactivation of the *Sepn1* gene in mice. *Sepn1^−/−^* mice were healthy and macroscopically indistinguishable from wild-type littermates. More surprisingly, we detected only subtle alterations in the morphology, ultrastructure and contractility of the mutant muscles. Together with our previous studies on embryonic development [20], these observations demonstrate that SelN is dispensable for survival, muscle development and muscle maintenance in mice, at least under basal housing conditions.

This absence of a major phenotype contrasts with the marked muscle defects observed in zebrafish morpholino mutants. In SelN-depleted zebrafish embryos, major developmental defects and important alteration of muscle architecture were observed [17], [22]. It was notably shown that SelN plays an important role for proper development of the slow muscle fiber lineage. This defect in the establishment and maintenance of slow fibers was not observed in the *Sepn1^−/−^* mouse. In addition, an alteration in the myoseptum structures was seen in mutant zebrafish, suggesting a possible function for SelN in the establishment of myotendinous junctions, the mammalian analogs of myosepta. This hypothesis could be excluded in our mouse model since EM analyses showed normal myotendinous junctions in all muscles examined (data not shown). In addition, a functional link between SelN and RyR was proposed, consistent with the alterations of calcium dynamics and triad structures detected in the zebrafish mutants [17]. Here we show that RyR1 and RyR3 expression and localization, as well as the structure of the triadic junctions, are not affected by SelN depletion in mice. RyR activity was not addressed in this study, but considering the preserved contractility of the *Sepn1^−/−^* muscles, it is unlikely that SelN deficiency drastically impairs RyR channel regulation, which would result in a severe perturbation of excitation-contraction coupling. One possible explanation is that the divergent phenotypes observed in zebrafish and mice may reflect existing differences in muscle development and physiology between the two species, and/or that SelN plays distinct functions in the two species. Similarly, the differences between the basal phenotype of our murine model and the symptoms described in *SEPN1*-RM patients is intriguing, but not exceptional: many mutations causing genetic neuromuscular diseases in humans lead to a mild phenotype once transposed to a mouse model [24], [25]. The absence of basal phenotype in the *Sepn1^−/−^* mice might result from the obvious differences existing between mice and human, including differential use of specific muscles, such as postural muscles. The human myopathy involves predominantly muscles which are constantly active, particularly postural back muscles whose tonic activity maintains the upright position, a situation clearly different for rodents. In addition, *SEPN1*-RM patients are ambulant and usually lead an active, close-to-normal life before the full phenotype develops in puberty [15]. In contrast, standard mouse housing conditions provide a stable environment, with restricted physical activity and controlled conditions of temperature or other potential stressors, which could contribute to protect these mice from spontaneous development of the phenotype.

Interestingly, while running tests failed to demonstrate or to induce any defects in SelN-deficient muscles, homozygous *Sepn1^−/−^* animals developed a pronounced phenotype when submitted to repeated forced swimming tests (FST). We decided to challenge our model with FST because this test associates physical exercise with a combination of behavior and environmental stresses. Indeed, FST is a widely used procedure to assess depression in mice, because it provides a context for global stress induction [26]–[28]. Moreover, during the swimming sessions, mouse back and neck muscles are strongly solicited, since they are submitted to constant isometric contractions in order to maintain the swimming posture. After repeated swimming sessions, *Sepn1^−/−^* mice developed symptoms reminiscent of the clinical spectrum described in *SEPN1*-RM patients, with a predominant effect on trunk muscles leading to a marked deformation of the spine and, at the cellular level, an abnormal switch toward smaller and slower fibers. Overall, this reveals a striking sensitivity of exercised SelN-deficient muscle to a recurrent stress context. In addition, our results demonstrate the important involvement of SelN in muscle physiology, since no other obvious defects were observed in any of the other organs analyzed.

As mentioned previously in patients, clinical signs are highly homogeneous and recognizable, but the severity and spectrum of histopathological presentations are variable [29]. In a recent publication, Cagliani and collaborators [14] described three families harboring *SEPN1* mutations with symptoms ranging from the severe early-onset rigid spine syndrome requiring early spinal fusion and assisted ventilation to more benign forms without major scoliosis. We also previously reported one patient with a mild phenotype first referred in her 30s, despite the almost complete absence of SelN expression [13]. This variability in the clinical severity, regardless of the nature and position of the mutation, suggests that other elements modulate the degree of the phenotype in humans; these could be unknown genetic variants but also non-genetic factors, in particular involvement of environmental and/or physiological stresses in the aggravation of the pathology. In this context, our animal model will be useful to evaluate the role of exercise and additional factors, such as nutritional or pharmacological, in the pathogenic mechanism of the disease.

We recently showed that SelN plays a major role in satellite cell maintenance in adult skeletal muscles, a mechanism essential for muscle regeneration under basal conditions and even more in an acute stress context, i.e. following cardiotoxin-induced necrosis/injury [30]. Hence, after two rounds of injury, a major impairment of the regeneration process was observed with drastic atrophy of the muscle. The absence of a basal phenotype in the *Sepn1^−/−^* mouse model indicates that this defect in satellite cell dynamics is nevertheless compatible with correct development, growth and maintenance of skeletal muscle tissues. In the FST conditions, we found that satellite cells were activated neither in wild-type nor in mutant mice (data not shown), suggesting that the abnormal phenotype developed by mutant mice is independent from the satellite cell defect. These observations suggest that SelN participates in at least two distinct (but not mutually exclusive) processes, namely, muscle progenitor maintenance and mature fiber homeostasis.

In the *Sepn1^−/−^* mouse model, the phenotype observed in the FST conditions developed after several repetitions of the swimming session, suggesting a cumulative process that progressively impairs muscle function. Previous studies based on *ex vivo* analyses of human cells indicated that SelN is involved in oxidative homeostasis in cells [18]. Accordingly, Oxyblot analysis revealed a significant increase in the overall content of carbonylated proteins in SelN-deficient quadriceps, but not in paravertebral muscles, under basal conditions (Figure S4). In the FST context, one explanation for the muscle alterations in mutants is that training induces an elevated tissue oxidation process, due to a combination of increased activity and stress. Interestingly, while FST induced an increase in oxidized protein content in quadriceps from both wild-type and mutant mice, in paravertebral muscles increased protein oxidation was observed in the mutant mice exclusively (Figure S4). This result suggested a different ability of mutant and wild-type paravertebral muscles to respond to stress and exercise. One remaining question is how the abnormal oxidative conditions resulting from FST would induce the alterations observed in the *Sepn1^−/−^* muscles, including the tubular aggregates and the fiber hypotrophy, notably in paravertebral muscles. It has been established that free radicals generated by oxidative stress in muscle disuse context participate in proteolysis induction and muscle atrophy [31], [32]. Moreover, disequilibrium in the redox potential within the muscle fiber could result in reticular stress and calcium homeostasis dysregulation [33], [34]. Thus, it seems possible that in the absence of SelN and in a situation of exercise and/or stress, altered oxidative conditions may lead to perturbed RyR activity. The subsequent altered calcium dynamics could then be responsible for atrophy, by activation of Ca^2+^-dependent proteases, and fiber type switch, by modifying the calcineurin pathway [35], [36]. Furthermore, it was shown that sarcoplasmic reticulum stress is responsible for tubular aggregate accumulation as an adaptive response to increased calcium influx [37]–[39].

Most members of the selenoprotein family, at least those with known functions, are enzymes involved in oxidation-reduction reactions and have been implicated in oxidative stress regulation [40]. Some selenoproteins, such as glutathione peroxidase 1 (GPx1), thioredoxin reductase (TrxR) and possibly selenoprotein P (SelP), are involved in the detoxification of reactive oxygen species. Others like methionine sulfoxyreductases (Msr) or phospholipid hydroperoxide glutathione peroxidase (GPx4) are involved in oxidative damage repair [41]. The molecular function of SelN is still elusive; whether it contributes to the maintenance of the redox homeostasis within the endoplasmic reticulum or controls more specific redox-regulated targets remains to be determined. One possible hypothesis, which we investigated in light of the mild muscle phenotype obtained in the *Sepn1^−/−^* mice, was a compensatory expression of other selenoprotein(s). Our data revealed no major change in the expression of selenoprotein transcripts, suggesting no functional redundancy between SelN and another protein of this family (Figure S1). Other enzymes or molecules involved in oxidative stress response may rather compensate the absence of SelN. One interesting candidate is vitamin E or α-tocopherol, a liposoluble antioxydant known to be involved in oxidative stress protection. Indeed, combined selenium and vitamin E nutritional deficiency has been linked to several muscle syndromes in both human and cattle [42]; reviewed in [6]. Moreover, *N*-acetylcysteine (NAC), a thiol donor that increases glutathione synthesis, was recently shown to reduce protein oxidation and protect SelN-deficient fibroblasts from cell death under oxidative stress conditions. Therefore, NAC has been envisioned as a potential treatment for individuals affected with *SEPN1*-RM [18]. The *Sepn1^−/−^* mouse model submitted to the exercise conditions described here will be useful to test this strategy.

In conclusion, we propose that despite the lack of a spontaneous phenotype, the *Sepn1* knock-out mice presented in this study constitute a useful tool to clarify the function(s) of SelN and to better understand the pathophysiological mechanisms underlying *SEPN1*-RM. While there was no obvious phenotype in normal housing conditions, we showed mild hypotrophy and absolute maximal force reduction in TA, limited fiber size modification in paravertebral muscles and an increase in oxidized protein levels; these data suggest that the pathophysiological process may already be on-going although not readily observable. In addition, specific experimental conditions demonstrated a particular susceptibility of *Sepn1* ^−/−^ muscles to exercise in a stress context, and led to a clear muscle phenotype whose distribution is comparable to that of *SEPN1*-RM patients. Therefore, further studies in this animal model will provide important clues to understand the influence of environmental factors in the disease pathogenesis, severity and progression, and might be helpful to identify and investigate potential therapeutic targets.

## Materials and Methods

### Generation of the *Sepn1* knock-out mice

Generation of the *Sepn1^−/−^* mice was performed in partnership with the Mouse Clinic Institute (Illkirch, France), according to previously described protocols [43]. Recombinant ES cell lines, derived from the 129 Sv Pas line [43], were screened by PCR to verify the correct recombination at the expected position within the genome, and Southern-blot analysis was carried out to ensure that the selected clones underwent a single and correct homologous recombination event. Genetically modified *Sepn1*-L_2_ and -L_3_ ES cell lines were injected into blastocysts arising from pseudo-pregnant C57/B6 J host females. Chimeric males were obtained for both transgenes and crossed with C57/B6 J females. The heterozygous offspring were then intercrossed to obtain homozygous (L_2_/L_2_) and (L_3_/L_3_) mice. For knock-out of the *Sepn1* gene, L_3_/L_3_ animals were bred with CMV-Cre mice. Constitutive expression of the Cre-recombinase in these mice led to the complete excision of both exon 3 and the Neo cassette, in particular within the germinal cell lineage and thus transmission of the disrupted gene (L-) to offspring. Heterozygous *Sepn1^+/−^* mice for the transgene were backcrossed to wild-type mice to remove the Cre gene. Intercrosses of heterozygous animals gave wild-type, heterozygous and knockout mice on a mixed C57/Bl6-129Sv background. Procedures involving animals and their care were conducted according to the European Union guidelines for animal care. Animal experimental procedures were evaluated and approved by CREMEAS (Comité Régional d'Ethique en Matière d'Expérimentation Animale de Strasbourg), number 2003-10-08[2], and by DDPP (Direction Départementale de la Protection des Populations), numbers D67-218-5 and 67-234. Genetically modified ES cell lines *Sepn1*-L2 and –L3, as well as the transgenique mouse *Sepn1*-K51 line were declared to Haut Conseil des Biotechnologies – Ministère de l′Enseignement Supérieur et de la Recherche, and registered under numbers 4465 of 26/05/2005 and 4712 of 26/05/2006.

### Forced Swimming Test (FST)

FST was performed as previously described [44]. Briefly, mice were submitted to FST in a plastic tank filled with 20 cm water, with no more than 3 mice per tank. Water temperature was maintained at 20°C. Mice were carefully placed into the water and their behavior was monitored. Mice were stimulated for active swimming by gentle pushes. The test was repeated every other day with a time increment of 1 min after each test (beginning at 6 min) until a swimming phase of 15 min was achieved over 3 months. At the end of each swimming session, mice were carefully dried and warmed under infrared light for 10 min.

### qRT-PCR analysis

Total RNA was extracted using the RNeasy Fibrous Tissue Mini Kit (Qiagen) according to the manufacturer's instructions. cDNA was synthesized from 500 ng of RNA with random hexamer primers using the SuperScript First-Strand Synthesis System for RT-PCR (Invitrogen). *Sepn1* transcripts were quantified as previously described [20]. Data were analyzed using the LightCycler480 analysis software (Roche). Primers used are listed in Table S1.

### Western blot analysis

Frozen tissues were ground in liquid nitrogen and homogenized in protein extraction buffer (80 mM Tris-HCl, pH 6.8, 10% SDS, 120 mM sucrose, 10 mM EDTA, 1 mM PMSF and 1 mM benzamidine). Homogenates were incubated for 10 min at 55°C, sonicated twice and quantified with the BCA Protein Assay (Pierce). 60 µg of proteins were electrophoretically separated on 8% polyacrylamide SDS gel and transferred to a polyvinylidene difluoride membrane (Invitrogen). Membranes were immunoprobed with rabbit anti-SelN (ab137) [16] and rabbit anti-actin (Sigma A2066) primary antibodies. Membranes were then incubated with HRP-conjugated secondary antibodies (Dako). Signals were detected with chemiluminescent HRP substrate (Immobilon Western - Millipore) on a G-Box-Chemimager (SynGene instrument, Ozyme).

### Muscle microsome preparation

RyR1, RyR3, DHPR and Trisk95 analysis were performed on membrane microsomes prepared from freshly dissected mouse skeletal muscles. Tissue was homogenized by dounce (1–2 g muscle/10 ml 200 mM sucrose, 20 mM HEPES pH 7.4, 0.4 mM CaCl_2_, 100 mM PMSF) and centrifuged at 1,400 g for 10 min. The resulting supernatant was centrifuged (41,000 g, 50 min), and the microsome pellet was resuspended in a 0.1 M NaCl, 30 mM imidazole pH 6.8, 8% sucrose buffer with protease inhibitors. Western blot analysis was performed as described above.

### Histological and ultrastructural analyses

Neural tube, eye, bladder, kidney, testis, intestine, stomach, liver, pancreas, brown fat, ligaments, bones and skin were analysed with standard histological staining performed on cryosections from E18 whole mouse embryo.

Muscles from *Sepn1^−/−^* and paired wild-type animals of three different age groups (n≥3 per group) were dissected and frozen in liquid nitrogen-cooled isopentane. Cryostat sections were stained with Hematoxylin/Eosin (HE), nicotinamide adenine dinucleotide-tetrazolium reductase (NADH-TR), succinate deshydrogenase (SDH), cytochrome c oxidase (COX), modified Gomori Trichrome (GT), periodic acid Schiff (PAS) and Oil Red O by classical methods (Dubowitzand and Sewry, 2007). Light microscopy observations were performed on quadriceps, tibialis anterior (TA), soleus, gastrocnemius, diaphragm and paravertebral muscles using an upright microscope (DMR, Leica) and 40× NA 0.85 HCX Plan Apo objective (Leica). Pictures were captured using a monochrome camera (DS-Ri1, Nikon) and NIS-Elements BR software (Nikon). Fiber size distribution was determined based on laminin immunostaining performed on muscle sections, using the NIS software (Nikon). Electron microscopy was performed on gastrocnemius, quadriceps or TA muscles first fixed with 2.5% glutaraldehyde in 0.1 M phosphate buffer (pH 7.4), and then with 2% OsO_4_ in 0.1 M phosphate buffer for 1 h at 4°C. Muscles were then dehydrated at 4°C in graded acetone including a 2% uranyl acetate in 70° acetone staining step, before Epon resin embedding. Thin (70 nm) sections were stained with uranyl acetate and lead citrate and observed using a Philips CM120 electron microscope (Philips Electronics NV) and photographed with a digital SIS Morada camera.

### Immunohistochemistry

The following antibodies were used on 8 µm muscle cryosections: embryonic MHC (DSHB, F1.652), slow MHC (DSHB, A4.840), fast MHC (DSHB, A4.74), DHPR, RyR3 (Chemicon, MAB427 and AB9082, respectively), laminin (Abcam, ab11575), RyR1 and Trisk95 antibodies (kind gift from Dr. Isabelle Marty, Grenoble Institut des Neurosciences, France). After microwave oven antigen retrieval, sections were blocked with 3% IgG free bovine serum albumin and ChromoPure Mouse IgG Fab Fragments (Jackson ImmunoResearch). Sections were incubated sequentially with primary antibodies and appropriate Alexa Fluor secondary antibodies (Invitrogen). They were mounted in Vectashield DAPI (Vector) and observed with an Axiophot microscope (Zeiss). Images were captured using the MetaView software (Ropper Scientific). RyR, DHPR and Trisk95 immunostainings were performed as previously described [45].

### Force measurement

The isometric contractile properties of TA muscles were studied *in situ*, as previously described [46]. Force measurements in diaphragm muscle were performed *in vitro*. Strips were dissected from diaphragm muscles *in situ* and placed in a dish containing oxygenated (95% O_2_ and 5% CO_2_) Tyrode's solution (0.2 g.L^−1^ CaCl_2_ 0.1 g.L^−1^ MgCl_2_ 0.2 g.L^−1^ KCl 8 g.L^−1^ NaCl 0.05 g.L^−1^ NaH_2_PO_4_, 1 g.L^−1^ D-Glucose and 0.1 g.L^−1^ NaHCO_3_, pH 7.4). Diaphragm muscle strips were tied firmly with surgical silk at the central tendon at one end and sutured through a portion of the rib attached to the distal end of the strip at the other end. Muscle strips were then transferred to a vertical bath filled with oxygenated Krebs-Ringer solution. The absolute maximal force (P_0_) was measured as described previously (Agbulut et al 2009). The muscle strips were weighted and the specific force sP_0_ was calculated as followed: sP_0_  =  P_0_ (N)/muscle mass (g) or P_0_ (N)/muscle area (mm^2^). Assuming muscles have a density of 1.06 mg/mm^3^, the cross-sectional area corresponds to the volume of the muscle divided by L_0_ (muscle length corresponding to maximal force). Fatigue resistance was determined after a 5 min resting period. Muscles were stimulated at 75 Hz during 500 ms every 2 s for 3 min, and the time taken for initial force to decrease by 30% (F30%) was determined.

### Images and Statistical analyses

The MetaMorph (Molecular Devices) and NIS (Nikon) software were used for cell counting and area measurement. Results are presented as means ± SEM of independent samples or animals; n represents the number of individual experiment (n≥3). Comparisons between groups were performed using the Student's *t*-test with a 0.05 level of confidence accepted for statistical significance.

## Supporting Information

Figure S1 **Selenoprotein expression in** ***Sepn1^−/−^*** **mice at E18.** Expression of eleven selenoproteins was quantified in E18 *Sepn1^+/+^* (black), *Sepn1^−/+^* (grey) and *Sepn1^−^* ^/*−*^ (white) embryos by qRT-PCR. For all of them, expression was unaltered in brain, quadriceps and diaphragm, with the exception of selenoprotein R (SelR), which was significantly increased in *Sepn1^−/−^*quadriceps. n = 3. *, p<0.05.
(TIF)

Figure S2 **Tomographic imaging shows an atrophic aspect of axial muscles of** ***Sepn1*** **^−/−^ mice after FST**. Coronal (A–C), sagittal (D–I) and transversal (J–O) sections of wild-type and *Sepn1^−/−^* 14 month-old mice submitted to FST; all corresponding sections are from equivalent body positions for the three animals. On coronal sections, *Sepn1^−/−^* mice (B, C) appeared leaner and emaciated compared to wild-type (A). Sagittal sections showed the diminution of the paravertebral muscle mass (arrows) in the *Sepn1^−/−^* mice submitted to FST (E, F). Moreover, increased curvature of the spine can clearly be observed on sagittal sections of the *Sepn1^−/−^* mice (E, F, H and I), this postural modification likely reflects the reduced tonicity of *Sepn1^−/−^* mice trunk muscles. The atrophy of the paravertebral muscles (circled with white dashed lines) is also observed on transversal sections at the pelvic level (K, L), while hind-limb muscles (delimitated by red dashed lines) appeared better preserved (see also sections C and D). In addition, transversal sections at the middle trunk level displayed reduction of the paravertebral mass in *Sepn1^−/−^* mice (compare N and O to M; paravertebral muscles are circled with white dashed lines).
(TIFF)

Figure S3 **Accumulation of triadic junction proteins DHPR, RyR1 and Trisk95 in the tubular aggregates of** ***Sepn1*** **^−/−^ mice muscles.** Immunostainings for DHPR (green) and RyR1 or Trisk 95 (red) on transversal sections of quadriceps from 14 month-old mice submitted to FST show that the tubular aggregates present in muscles from *Sepn1^−/−^*, but not from wild-type mice, were highly enriched in triadic junction proteins, in agreement with a SR origin of these structure previously reported in the literature [36].
(TIF)

Figure S4 **Oxyblot analysis of quadriceps and paravertebral muscles of** ***Sepn1*** **^+/+^ and** ***Sepn1*** **^−/−^ mice before and after FST.** Histograms represent mean values of total carbonyl optical densities in quadriceps and paravertebral muscles from 14 month-old *Sepn1* ^+/+^ and *Sepn1* ^−/−^ mice, subjected or not to FST. n = 3. # p<0.05 between *Sepn1* ^+/+^ and *Sepn* ^−/−^ muscles; * p<0.05 between basal and FST-stressed muscles.
(TIF)

Table S1 **Primers used for qPCR analysis.** Primer sequences are shown in their 5′ to 3′ orientation.
(DOC)

Movie S1 **Forced Swimming Test (FST).** **Sequence 1)**. General overview of the swimming test device. **Sequence 2) Wild-type FST training session**. Five month-old wild-type mice are actively swimming at the second week of FST. **Sequence 3)** ***Sepn1^−/−^*** **FST training session**. Five month-old *Sepn1^−/−^* are actively swimming at the second week of FST exercise, similarly to wild-types. **Sequence 4)** ***Sepn1^−/−^*** **FST week 10**. While 6 month-old wild-types are still actively swimming after 10 weeks, *Sepn1^−/−^* mice of the same age showed a marked reduction of motility, accompanied by a global rigidity. **Sequence 5)** ***Sepn1^−/−^*** **Immediately post-FST**, **week 10**. Once taken out of the water, 6 month-old *Sepn1^−/−^* mice, which have been practicing FST for 10 weeks, remained stiff with a global body and limbs rigidity. This rigidity lasted for several minutes, before the mice recovered their mobility, a behavior that was not observed in wild-types. **Sequence 6) Spontaneous activity of wild-type mice after 10 weeks FST**. Six month-old wild-type mice that have been subjected to FST for 10 weeks are shown for comparison with **Sequence 7)** ***Sepn1^−/−^*** **spontaneous activity post 10 weeks FST**. Although 6 month-old *Sepn1^−/−^* mice subjected to FST for 10 weeks showed normal mobility, they displayed a clear deformation of the back. **Sequence 8) Comparison between** ***Sepn1^−/−^*** **and wild-type, post 14 weeks FST**. 14 month-old mutant or wild-type mice practicing FST for 14 weeks. The mutant mouse can easily be distinguished, because of the severe kyphosis. Note that despite the deformation of the back, the SelN-deficient mouse remained active.
(MOV)

Data S1 **Treadmill running test.** The purpose of this experiment was to analyze the ability of *Sepn1^−/−^* mice to adapt to an exercise-training test compared to wild-type controls. Training consisted of running on a motorized treadmill (Colombus Instrument) at 0% gradient at 15 m/min 3 sessions/week for 20 min the first week, 30 min the second week and 40 min the third week. After a 15 min acclimation period without motion, the mice were warmed-up by increasing speed from 9 to 15 m/min during the initial 3 min (acceleration 2 m/min^2^). Running was stimulated using electrical shock grids. Transgenic mice adapted as well as the wild-types and were able to complete the running tests without developing any characteristic feature. **Detection of Protein Carbonyls**. Carbonylated proteins were detected using immunoblotting (OxyBlot Protein Oxidation Detection; Invitrogen) as described previously [47] and a HRP chemiluminescent detection (Millipore). Muscle protein samples without the derivatization step were used as negative controls. After scanning with an imaging densitometer, optical densities (OD) of protein bands were quantified using the Image J software. Total protein carbonylation OD in a given sample was calculated by adding OD of individual carbonylated protein bands.
(DOC)
