# Supplementary figures and images for: Increased Muscle Stress-Sensitivity Induced by Selenoprotein N Inactivation in Mouse: A Mammalian Model for SEPN1-Related Myopathy
Source: PLoS One. 2011 Aug 8;6(8):e23094. doi: 10.1371/journal.pone.0023094 (PMC3152547; doi:10.1371/journal.pone.0023094)

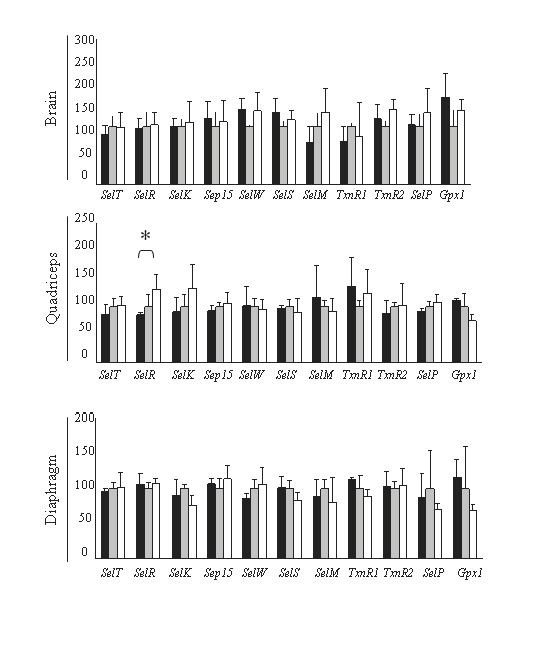

Supplement: Figure S1 — Selenoprotein expression in Sepn1−/− mice at E18. Expression of eleven selenoproteins was quantified in E18 Sepn1+/+ (black), Sepn1−/+ (grey) and Sepn1− /− (white) embryos by qRT-PCR. For all of them, expression was unaltered in brain, quadriceps and diaphragm, with the exception of selenoprotein R (SelR), which was significantly increased in Sepn1−/−quadriceps. n = 3. *, p<0.05. (TIF) [file pone.0023094.s001.tif]

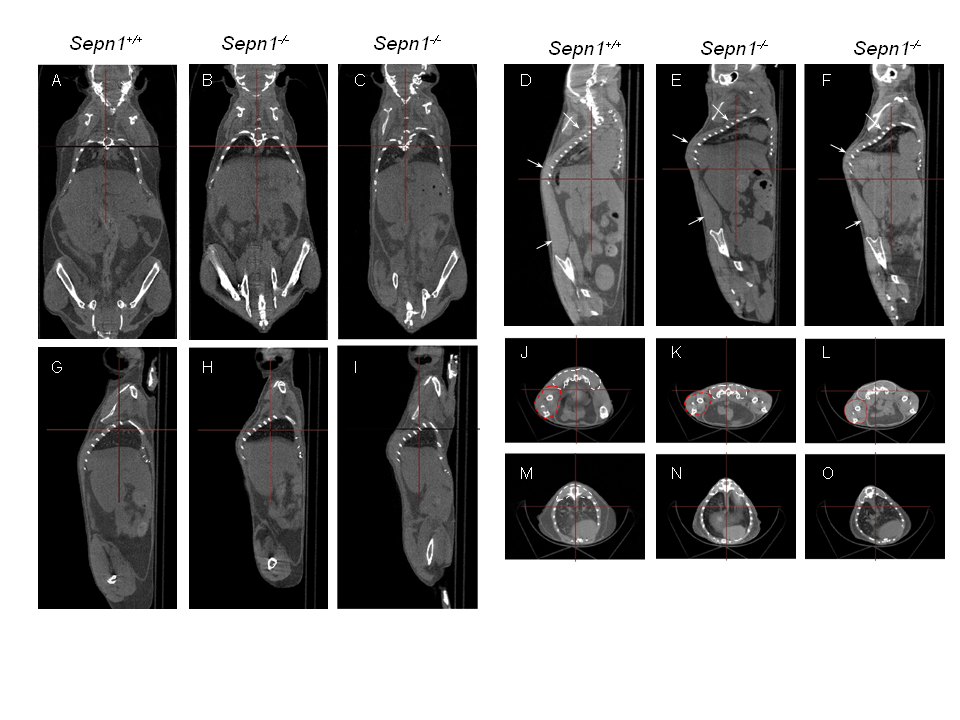

Supplement: Figure S2 — Tomographic imaging shows an atrophic aspect of axial muscles of Sepn1 −/− mice after FST. Coronal (A–C), sagittal (D–I) and transversal (J–O) sections of wild-type and Sepn1−/− 14 month-old mice submitted to FST; all corresponding sections are from equivalent body positions for the three animals. On coronal sections, Sepn1−/− mice (B, C) appeared leaner and emaciated compared to wild-type (A). Sagittal sections showed the diminution of the paravertebral muscle mass (arrows) in the Sepn1−/− mice submitted to FST (E, F). Moreover, increased curvature of the spine can clearly be observed on sagittal sections of the Sepn1−/− mice (E, F, H and I), this postural modification likely reflects the reduced tonicity of Sepn1−/− mice trunk muscles. The atrophy of the paravertebral muscles (circled with white dashed lines) is also observed on transversal sections at the pelvic level (K, L), while hind-limb muscles (delimitated by red dashed lines) appeared better preserved (see also sections C and D). In addition, transversal sections at the middle trunk level displayed reduction of the paravertebral mass in Sepn1−/− mice (compare N and O to M; paravertebral muscles are circled with white dashed lines). (TIFF) [file pone.0023094.s002.tiff]

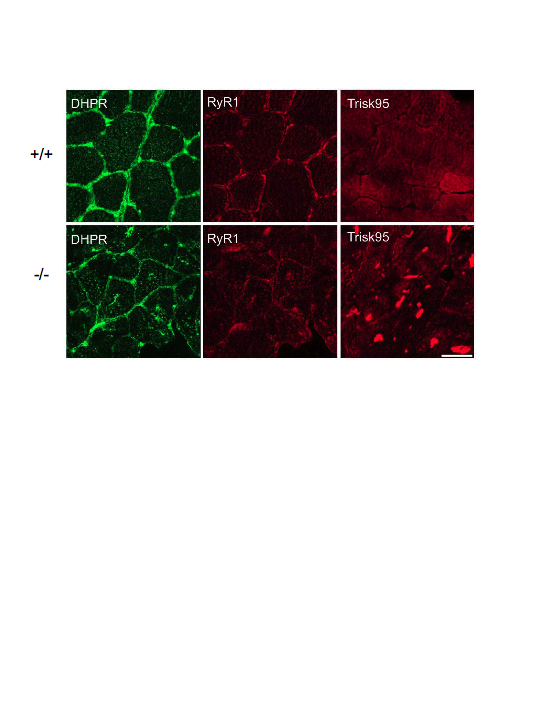

Supplement: Figure S3 — Accumulation of triadic junction proteins DHPR, RyR1 and Trisk95 in the tubular aggregates of Sepn1 −/− mice muscles. Immunostainings for DHPR (green) and RyR1 or Trisk 95 (red) on transversal sections of quadriceps from 14 month-old mice submitted to FST show that the tubular aggregates present in muscles from Sepn1−/−, but not from wild-type mice, were highly enriched in triadic junction proteins, in agreement with a SR origin of these structure previously reported in the literature [36]. (TIF) [file pone.0023094.s003.tif]

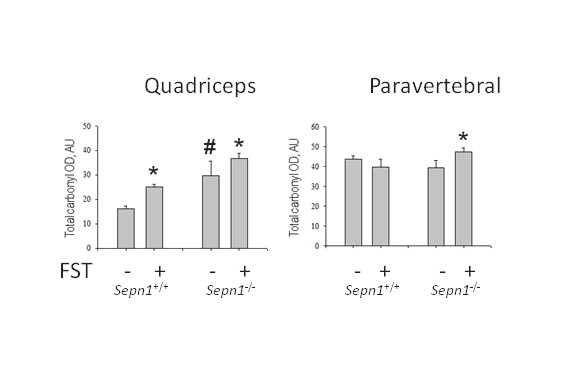

Supplement: Figure S4 — Oxyblot analysis of quadriceps and paravertebral muscles of Sepn1 +/+ and Sepn1 −/− mice before and after FST. Histograms represent mean values of total carbonyl optical densities in quadriceps and paravertebral muscles from 14 month-old Sepn1 +/+ and Sepn1 −/− mice, subjected or not to FST. n = 3. # p<0.05 between Sepn1 +/+ and Sepn −/− muscles; * p<0.05 between basal and FST-stressed muscles. (TIF) [file pone.0023094.s004.tif]
