# Supplementary material for: Increased Muscle Stress-Sensitivity Induced by Selenoprotein N Inactivation in Mouse: A Mammalian Model for SEPN1-Related Myopathy
Source: PLoS One. 2011 Aug 8;6(8):e23094. doi: 10.1371/journal.pone.0023094 (PMC3152547; doi:10.1371/journal.pone.0023094)
Supplement: Table S1 — Primers used for qPCR analysis. Primer sequences are shown in their 5′ to 3′ orientation. (DOC) [file pone.0023094.s005.doc]

**Table S1. Primers used for qPCR analysis.** Primer sequences are shown in their 5’ to 3’ orientation.

| selenoprotein | Forward primer | Reverse Primer |
| --- | --- | --- |
| SelN | GCTTTCCTGTAGAGATGATG | GCCCCGCCGGAGTCCTTC |
| SelT | tatccagacatccgcattga | ttattttcttggccccactg |
| MsrB | acagttgttgccccattagc | ggagtgggtctcagcttcag |
| SelK | gctggtggatgaggaaggta | caggctagatcctgcagagg |
| Sep15 | Ggatgaaaattggggaggtt | Gttccaacttctcgctcagg |
| SelW | Cccaagtacctccagctcaa | ttccggaacttgctctctgt |
| SelS | tggcggctgaaactaagact | gtggcctaatggcaatgtct |
| SelM | ccgaggaagcttcagaacac | Ggagaggaaagggaggattg |
| Txn1 | Tggatccatttccatctggt | ccacaccacgtagcagagaa |
| Txn2 | gcttctggcaaggaagacac | Ccctcagcaacatctccaat |
| Sepp | gagggcaaagtgacagtggt | tgggaaggagatccttgatg |
| Gpx1 | aaggtggattgcaacaaagg | Caaaaccgtgacgttgaatg |
