# Supplementary material for: Increased Muscle Stress-Sensitivity Induced by Selenoprotein N Inactivation in Mouse: A Mammalian Model for SEPN1-Related Myopathy
Source: PLoS One. 2011 Aug 8;6(8):e23094. doi: 10.1371/journal.pone.0023094 (PMC3152547; doi:10.1371/journal.pone.0023094)
Supplement: Data S1 — Treadmill running test. The purpose of this experiment was to analyze the ability of Sepn1−/− mice to adapt to an exercise-training test compared to wild-type controls. Training consisted of running on a motorized treadmill (Colombus Instrument) at 0% gradient at 15 m/min 3 sessions/week for 20 min the first week, 30 min the second week and 40 min the third week. After a 15 min acclimation period without motion, the mice were warmed-up by increasing speed from 9 to 15 m/min during the initial 3 min (acceleration 2 m/min2). Running was stimulated using electrical shock grids. Transgenic mice adapted as well as the wild-types and were able to complete the running tests without developing any characteristic feature. Detection of Protein Carbonyls. Carbonylated proteins were detected using immunoblotting (OxyBlot Protein Oxidation Detection; Invitrogen) as described previously [47] and a HRP chemiluminescent detection (Millipore). Muscle protein samples without the derivatization step were used as negative controls. After scanning with an imaging densitometer, optical densities (OD) of protein bands were quantified using the Image J software. Total protein carbonylation OD in a given sample was calculated by adding OD of individual carbonylated protein bands. (DOC) [file pone.0023094.s007.doc]

**Data S1.**

**Treadmill running test.** The purpose of this experiment was to analyze the ability of *Sepn1^-/-^* mice to adapt to an exercise-training test compared to wild-type controls. Training consisted of running on a motorized treadmill (Colombus Instrument) at 0% gradient at 15 m/min 3 sessions/week for 20 min the first week, 30 min the second week and 40 min the third week. After a 15 min acclimation period without motion, the mice were warmed-up by increasing speed from 9 to 15 m/min during the initial 3 min (acceleration 2 m/min^2^). Running was stimulated using electrical shock grids. Transgenic mice adapted as well as the wild-types and were able to complete the running tests without developing any characteristic feature.

**Detection of Protein Carbonyls.** Carbonylated proteins were detected using immunoblotting (OxyBlot Protein Oxidation Detection; Invitrogen) as described previously [47] and a HRP chemiluminescent detection (Millipore). Muscle protein samples without the derivatization step were used as negative controls. After scanning with an imaging densitometer, optical densities (OD) of protein bands were quantified using the Image J software. Total protein carbonylation OD in a given sample was calculated by adding OD of individual carbonylated protein bands.
